# Attempt to increase the transparency of fourth hurdle implementation in Central-Eastern European middle income countries: publication of the critical appraisal methodology

**DOI:** 10.1186/1472-6963-12-332

**Published:** 2012-09-21

**Authors:** András Inotai, Márta Pékli, Gabriella Jóna, Orsolya Nagy, Edit Remák, Zoltán Kaló

**Affiliations:** 1 University Pharmacy, Department of Pharmacy Administration, Semmelweis University, Hőgyes E. u. 7-9, Budapest, 1092, Hungary; 2 Office of Health Technology Assessment, National Institute for Quality- and Organizational Development in Healthcare and Medicines, Diós árok 3, Budapest, 1125, Hungary; 3 United BioSource Corporation, Bég u 3-5, Budapest, 1022, Hungary; 4 Health Economics Research Centre, Eötvös Loránd Science University, Pázmány P. 1a, Budapest, 1117, Hungary; 5 Syreon Research Institute, Thököly út 119, Budapest, 1146, Hungary

## Abstract

**Background:**

In middle income countries the number of trained health technology assessment specialists is limited and the public budget for health technology assessment is considerably lower compared to developed countries. These countries therefore must develop their own solutions to improve the quality and efficiency of health technology assessment implementation in reimbursement decisions. Our study aimed to develop a scientifically rigorous and detailed appraisal checklist for economic evaluations of pharmaceuticals in the single health technology assessment process.

**Methods:**

The research design entailed a review of economic evaluations, submitted for reimbursement of pharmaceuticals, by two independent academic reviewers to identify the most common methodological problems. Fifty economic evaluations submitted in 2007-2008, randomly selected by the Health Technology Assessment Office served as data sources. The new checklist was developed by an iterative working process: first by assessing ten economic evaluations, then improving the checklist by generating new question items, then employing the improved checklist to assess the next ten economic evaluations. After appraising 25 documents, the reviewers reconciled their opinions and improved the checklist with the researchers of the Health Technology Assessment Office during an expert panel discussion. The reviewers scrutinized the second 25 economic evaluations, after which the expert panel finalized the checklist with consensus.

**Results:**

The final checklist consists of 91 yes or no questions in 11 main topics concerning comparator selection, efficacy, effectiveness, costs, sensitivity analysis, methodological approach, transparency, and interpretation of results. The new checklist is based on current Hungarian evaluation practice. As the published checklist will be part of the official single health technology assessment process of pharmaceuticals, submitters will be able to assure the quality of their economic evaluation.

**Conclusions:**

The transparent critical appraisal method should improve the consistency of pharmaceutical reimbursement decisions and facilitate the utilization of economic evaluations in other fields of health care decision-making in other Central-Eastern European countries.

## Background

Compared to high-income European countries, middle income Central and Eastern European (CEE) countries have worse health status [1,2]. To improve the health of population, these countries have even more limited resources than Western European countries. As the value based price of innovative health technologies is adjusted to top 5 European countries, the price is often relatively too high in countries with lower income [3]. Consequently the need for justification of cost-effectiveness for expensive technologies is even greater in CEE countries. Unfortunately the number of trained health economists and health technology assessment (HTA) specialists is limited and the public budget for HTA is lower than in developed countries [4-6]. Therefore middle income countries must develop their own solutions to improve the quality and efficiency of HTA implementation in reimbursement decisions.

The first necessity is a development of a guideline for economic evaluations. It is, however, difficult to develop guideline which is perfectly relevant for all different kind of health technologies and services. For example, the HTA approach for medical devices is different from pharmaceuticals due to the limited availability of randomised controlled trials (RCTs), and other methodological problems such as multiple indications, frequent product modifications, learning curves, and high fixed cost [7]. HTA guidelines are usually broad enough to cover all different aspects [8,9], however the National Institute for Health and Clinical Excellence also developed focused guidelines for diagnostics [10] and devices [11].

The second necessity of quality assurance in HTA is the use of critical appraisal checklists especially in those countries where single HTA process is implemented. In the current examples of this process this means that the manufacturer of the technology prepares the assessment of the technology. The public HTA office cannot prepare an independent assessment (double technology assessment) and is only responsible for the critical appraisal of the submitted material. In countries with single HTA process, the most critical question is how to ensure the appropriateness, the homogeneity and the transparency of the critical appraisal. There are two different approaches: The first focuses on the transparent description of the appraisal methodology, the second focuses more on disclosing details of each individual case. As submissions may contain confidential information (i.e. proposed price of the technology), the first approach can be easily implemented in any countries.

There are many available international checklists [12-15], however, they are often not detailed enough to address specificities of long HTA submissions or not adjusted to the country specific methodological problems. Therefore authors suggest that for critical appraisal of single technology assessment, detailed country and technology specific checklists should be developed based on the review of existing local HTA practices.

In Hungary cost-effectiveness and budget impact analyses have been mandated in the reimbursement process of new pharmaceuticals since 2003. Guidelines for economic evaluations were published [16] in 2002, but have never been updated. These guidelines covered all health care interventions therefore they were not specific for pharmaceuticals and not targeted to reimbursement questions. The first six years experience of mandatory fourth hurdle (i.e. local cost-effectiveness evidence) for new pharmaceuticals indicated that the quality of economic evaluations submitted in pharmaceutical reimbursement dossiers and the quality of critical appraisal by the HTA Office varied considerably. Similar trends have been observed in Canada [17], Latin America [18], and Sweden [19].

Therefore in 2009, an expert panel was established to develop a detailed, publicly available, scientifically rigorous, and policy-relevant Hungarian critical appraisal checklist to improve the quality of economic evaluations and budget impact analyses submitted for single health technology assessment in pharmaceutical reimbursement applications and to reduce the heterogeneity of appraisals. This paper describes the methodological approach of checklist development, and presents the final checklist.

## Methods

**Figure 1:**
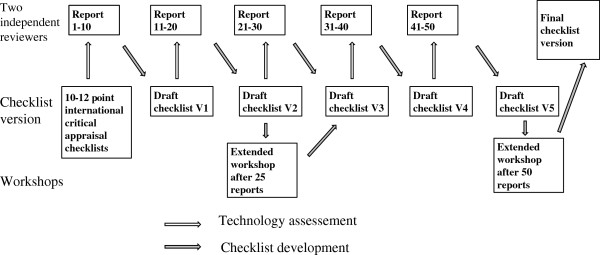
Development of the checklist.

The expert panel consisted of two independent academic experts, who scrutinized previous submissions and developed a new draft checklist, and members of the HTA Office who supervised the project and approved new items in the checklist. To maintain the full support of decision-makers in the Ministry of Health and the National Health Insurance Fund the project had no intention to revise or comment on previous reimbursement decisions. Overall, 50 consecutive economic evaluations that had been submitted for reimbursement of pharmaceuticals in 2007-2008 were selected by the HTA Office to be scrutinized by the two independent academic reviewers for the most common methodological problems. The first ten submissions were assessed using the previously translated Critical Appraisal Skills Programme (CASP) questionnaire [20] in the form of a spreadsheet independently completed by each reviewer. In the process, several new items were added to reflect common methodological or technical problems. The spreadsheet included a column listing the questions from the new checklist (one per row), and each economic evaluation had a separate column to record the reviewers’ answers and comments. The assessments of the first ten submissions were then reconciled by the reviewers. The format of the CASP checklist was also changed; as many of the new question items were not relevant for all submitted economic evaluations, a “not relevant” option was added to “yes” or “no” option. For the next ten submissions, a draft version of the new extended checklist was employed (Figure 1). Each new technical problem observed generated a new question item under the assumption that these questions could prevent future occurrences of these problems. Consequently, the development process was iterative: as another generally applicable problem was identified the reviewers added a new question item to the draft questionnaire. After 25 economic evaluations were assessed, the opinions of the reviewers were again reconciled and the checklist was revised through an expert panel discussion with the entire staff of the HTA Office. Subsequently, the reviewers scrutinized the second 25 economic evaluations, again extending the checklist with further question items. The final checklist was achieved through a consensus decision of the expert panel.

The Hungarian Critical Appraisal Checklist is not a scorecard; it only lists the most relevant questions for facilitating the appraisal process. While quantitative questionnaires (i.e., those with scoring) can be validated by statistical methods, it is less obvious how to validate a qualitative critical appraisal checklist. The development process described here incorporated three steps to improve validity. First, every new question item was incorporated only through consensus of the expert panel. Second, in a 14-month probation period, between September 2009 and November 2010, the checklist was used by the public HTA office in the appraisal of 80 new pharmaceutical reimbursement submissions. Researchers of the HTA Office confirmed the appropriateness of the checklist and reported no need for further amendments. Finally, an independent opponent was given the opportunity to publicly challenge the pre-circulated checklist during a two-hour meeting of the Hungarian Health Economics Association dedicated to the public discussion of the checklist. This followed an approach similar to that of the Health Economist’s Study Group in the United Kingdom [21].

## Results

The final Hungarian Critical Appraisal Checklist (Additional file 1) has two major themes: the first addresses economic evaluations, whereas the second concentrates on budget impact analyses. Economic evaluations are the subject of 80 yes or no questions in ten major sections; 11 questions relate specifically to budget impact analysis (see Table 1).

**Table 1:** Structure of the checklist

	**Topic**	**# of items**
1.	Economic Evaluation	80
1.1.	Filter Questions	2
1.2.	Research Question (relevance, comparator, financing protocol)	2
1.3.	Health Benefit	25
1.3.1.	Source of Scientific Evidence	7
1.3.2.	Evaluation of Relative Effectiveness in Case of Indirect Comparison	10
1.3.3.	Magnitude of Health Benefit	8
1.4.	Cost	8
1.5.	Time Horizon, Discounting	3
1.6	Alternative Sections for Methodology	22
1.6.1.	Cost-Minimization Analysis	3
1.6.2.	Cost Effectiveness Analyses	19
1.6.2.1.	Decision Tree Model	4
1.6.2.2.	Markov Model	7
1.6.2.3.	Simulation Model	8
1.7.	Decision Rule	3
1.8.	Sensitivity Analysis	6
1.9.	General Methodology: Adequacy and Transparency	5
1.10.	Interpretation regarding the Economic Evaluation	4
2.	Budget Impact Analysis	11
	Total checklist	91

The ten sections addressing economic evaluations cover core ideas related to the selection of comparators, effectiveness, costs, sensitivity analysis, methodological approach, transparency, and interpretation of results. To reflect the possible variations in study design of economic evaluations, separate alternative sections were developed for cost-minimization analyses and modeling approaches of cost-effectiveness analyses (including decision tree, Markov and simulation models). This separation allows the incorporation of methodology-specific issues into the checklist. Interestingly, none of the 50 reimbursement dossiers reviewed included economic analyses conducted alongside prospective clinical trials; this was mainly due to the fact that Hungary is too small market for conducting adequately powered prospective trials for local reimbursement purposes.

When the checklist is used for critical appraisal, reviewers may exclude non-relevant question items. If the problem raised by a question is relevant and has been addressed appropriately in the economic analysis of pharmaceuticals, the question should be answered “yes”. If a “yes” answer cannot be justified in the case of a relevant question, the response should be “no”. The authors therefore encourage the inclusion of explanatory comments for all answers in the “explanation” column. Sub-questions in brackets that follow some questions also prompt explanations.

An important issue when developing checklists is the distinction between the positive aspect of reporting (e.g. did they apply discounting?) and normative aspects of methods (e.g. did they discount by a rate of 5% per annum in real terms?). We took an intermediate approach, in several cases we considered the normative aspects without clarifying what is the only appropriate approach (e.g. did they apply the appropriate discount rate?), as the correct approach can be different for particular technologies.

In use, a higher proportion of “yes” answers would indicate the appropriateness of the economic evaluation and budget impact analysis for inclusion in the reimbursement dossier. However, the questions are not equal and not weighted; therefore, the critical appraisal checklist cannot transform the assessment of the quality of the single technology appraisal into an automatic process. There are twofold benefits from the checklist. At first to guide those who prepare submissions on what are the most critical methodological and technical questions of the HTA report, secondly to make sure that these important factors are not missed in the critical appraisal process, therefore the heterogeneity of appraisals is reduced.

## Discussion

Certain countries assure the quality of HTA in the reimbursement decision process by improving the details of their guidelines; however in these cases the guideline may not be relevant for all different technologies (e.g. pharmaceuticals, medical devices, screening procedures etc.). For middle income CEE countries with limited budget and capacity, general guidelines and detailed supplementary checklists for each kind of health technologies could be an optimal solution. As a first step, we presented a prototype how to prepare such a scientifically rigorous appraisal tool specifically developed for pharmaceutical technologies based on the quality assessment of economic evaluations in previous reimbursement dossiers. The existence of such tool, however, could not substitute the overdue updating of the Hungarian guidelines for economic evaluation or the development of a Reference Guide to those making submissions. Development of similar checklists for medical devices or other medical technologies should also be considered. Both the methodology of development and/or the actual questions of the checklist might be of interest for other CEE countries with single HTA to assure the better use of economic evaluations in the reimbursement of pharmaceuticals. For CEE countries where HTA was recently introduced, this checklist could serve as a draft to develop their own country specific questionnaire, with the adaptation of the iterative methodology described in this paper.

This Hungarian checklist was developed to detect and consequently to prevent potential methodological and technical problems seen in economic evaluations in pharmaceutical reimbursement submissions. Identification of these issues requires advanced skills; thus, authors want to emphasize how important the qualifications of the assessors are [22]. However, based on the 14 month probation period, such a detailed checklist is a useful aid even for new and relatively inexperienced associates of the HTA Office.

There is a trade-off between the sensitivity and simplicity of critical appraisal checklists (Table 2 compares some well known international checklists with the Hungarian Critical Appraisal Checklist). Shorter questionnaires are suitable for a rapid assessment of economic evaluations, e.g., a review of scientific papers [23]. However, neither the shorter [24] nor the longer [15] international critical appraisal checklists are detailed enough to reliably filter all problems observed in health economic analyses in reimbursement submissions of pharmaceuticals, particularly as inappropriate decisions by the authorities may result in serious health and financial consequences. Furthermore, the more general questions seen on the shorter lists can be interpreted differently, further eroding their usefulness for assessors. The majority of existing questionnaires are based on the current sophisticated skill set and practice of developed countries, therefore may not be suitable in middle income countries due to limited capacity of experts and heterogeneity of submissions. The time required to answer a detailed questionnaire (such as the Hungarian Critical Appraisal Checklist with 91 items) must be acknowledged, but the thorough revision of data and methodology in a public HTA appraisal requires several days for each submission anyway. In addition, compared with scientific publications, economic evaluations in reimbursement submissions are lengthier, and include more details about assumptions, input data, and study methodology [25].

**Table 2:** Comparison of the Hungarian Critical Appraisal Checklist to some other international checklists

**Name of the checklist**	**Drummond/CASP checklist**	**British Medical Journal (BMJ) checklist**	**Consensus Health Economic Criteria (CHEC) list**	**Quality of Health Economic Studies (QHES) grading system**	**Hungarian Critical Appraisal Checklist**
Number of questions	10 / 12	35	19	16	91
Answer options	YES/NO/Can't tell	YES/NO/Not appropriate	YES/NO	YES/NO	YES/NO/Not relevant
Quantitative assessment (including weighting different items) available?	no	no	no	yes	no
Reference	[20,24]	[15,22]	[14,22]	[13,22]	NA.

Some unique items of the Hungarian Critical Appraisal Checklist should be highlighted. Specific questions are dedicated to therapeutic guidelines, the financing protocol (i.e., whether the therapy is reimbursed for first-line use or only second- or third-line), and the level of reimbursement (due to the complexity of the Hungarian public financing system). Such questions were not common in previous checklists. Based on 50 submissions, a question is dedicated to prevent inappropriate selection of studies favouring the investigational technology. Assessment of relative effectiveness in indirect comparison of pharmaceuticals has gained increasing importance recently [26], as the comparator in pivotal clinical trials may not be policy relevant in all countries, e.g., the comparator may not be reimbursed or widely used. As a consequence, ten specific questions have been included to evaluate the methodology of relative effectiveness in case an indirect comparison is employed in submissions. A recurring methodological error was the calculation of daily therapeutic drug cost from the perspective of the third-party payer. Unless the full public price is employed in the calculation, the most cost-effective scenario from the payer’s perspective would be a 0% reimbursement, i.e., no increase in drug costs with additional health gain. By requesting the graphical structure and detailed description of decision tree and Markov models and the transparent description of all input data, the HTA Office should be able to reconstruct some of the economic models [27]. This practice prevents the assessment of cost-effectiveness based on black box economic models, which was common approach previously. In some extreme submissions, a long general introduction was accompanied by a brief economic evaluation; therefore, a question was dedicated to address the proportionality of economic evaluation. Explicit declaration of known limitations in the submitted economic evaluation is also not a regular part of critical appraisal checklists; the Hungarian checklist addresses this issue. According to Ramsberg et al. [19], checklists tend to be too general to pick up fine distinctions of specific models.

A limitation of the Hungarian Critical Appraisal Checklist could be that its development is based on the reappraisal of only 50 submissions, and thus may not reflect all potential problems. The Hungarian checklist does not employ a scoring system; consequently it cannot rank different economic evaluations quantitatively. Unless items are explicitly weighted, the implicit general assumption is that all questions are treated as equally important [28]. Compared with previous checklists with a scoring system, the Hungarian Critical Appraisal Checklist, as a qualitative tool, could not be validated with statistical methods. Certain steps of the methodological approach employed in this research were similar to methods in previous publications. Gerkens et al. [22] appraised nine economic evaluations to compare three different checklists (British Medical Journal (BMJ) checklist, Consensus Health Economic Criteria (CHEC) list, Quality of Health Economic Studies (QHES) grading system). The development of a scoring system by Gonzalez-Perez [23] was based on a 50-study sample. Ramsberg et al. [19] scrutinized a sample of 20 submissions in Sweden (2002-2003). Similarly to our approach, these studies applied at least two independent reviewers.

A further development of the Hungarian checklist could be to assign weights to each question. However, the aim of this checklist is not to score submissions by counting the number of “yes” answers, but to avoid methodological and technical errors in future submissions by using this instrument when compiling pharmaceutical reimbursement dossiers. Although the checklist is based on current Hungarian practice, the authors could dedicate questions only to those problems that occurred in the 50 assessed HTA documents. The authors recommend that the checklist be revised periodically, approximately every two years. Continuous development of the checklist can prevent to be grounded in the current methodology. E.g. the current methodological guidelines for economic evaluations in Hungary does not necessitate the use of probabilistic sensitivity analysis (PSA), once it becomes recommended, the checklist can be extended with new items on PSAs. As the Hungarian methodological guidelines determine normative aspects of economic analyses and some of these items (e.g. discount rate, cost effectiveness threshold) are subject to change in the forthcoming guidelines, the critical appraisal checklist does not mention any normative aspects to ensure full harmonisation with the guidelines.

## Conclusions

The new Hungarian Critical Appraisal Checklist is detailed enough to address the most common problems in local economic evaluations and budget impact analyses submitted in reimbursement dossiers by pharmaceutical companies. The published checklist will be used officially by the HTA Office in the pharmaceutical’s single health technology assessment process. As it will be in the public domain, application of the checklist should improve the consistency of the appraisal process and consequently encourage pharmaceutical companies to assure the quality of their submitted economic evaluations. Overall, the use of a transparent method of single technology assessment should improve the appropriateness of pharmaceutical reimbursement decisions.

The authors believe that the Hungarian Critical Appraisal Checklist will prove to be a significant step towards the better use of economic evaluations in the reimbursement of pharmaceuticals and by adopting the development methodology or certain questions, may have policy implications for several other Central-Eastern European middle income countries. It is noteworthy, especially in Central-Eastern Europe, that a public authority has been willing to develop and publish scientifically rigorous criteria for decision-making purposes. We are not aware of any published critical appraisal checklists specifically developed for pharmaceuticals based on the HTA practice and skill set of middle income countries.

## Competing interests

The authors declare that they have no competing interests.

## Authors’ contributions

AI. independent academic reviewer nr.1, draft checklist development, draft article, corresponding author MP. head of expert panel (HTA office), supervisor of the new checklist, article revision GJ. expert panel member (HTA office), supervisor of the new checklist, article revision ON. expert panel member (HTA office), supervisor of the new checklist, checklist and article revision ER. simulation model checklist questions, expert panel member, article revision ZK. independent academic reviewer nr.2, scientific guarantor, draft checklist development, article revision and approval. All authors read and approved the final manuscript.

## Pre-publication history

The pre-publication history for this paper can be accessed here:

http://www.biomedcentral.com/1472-6963/12/332/prepub

## Supplementary Material

Additional file 1 Hungarian critical appraisal checklist. Click here for file

## References

[B1] Szalay T Pazitný P Szalayová A Frisová S Morvay K Petrovic M van Ginneken E Slovakia health system review Health Syst Transit 2011 13 1 174 21540135

[B2] OECD Health at a Glance 2011 http://www.oecd.org/dataoecd/6/28/49105858.pdf Accessed [14 May 2012]

[B3] Inotai A Kaló Z Risk sharing methods in middle income countries Acta Pharm Hung 2012 82 43 52 Hungarian 22570986

[B4] Tantivess S Teerawattananon Y Mills A Strengthening cost-effectiveness analysis in Thailand through the establishment of the health intervention and technology assessment program Pharmacoeconomics 2009 27 931 945 10.2165/11314710-000000000-00000 19888793

[B5] Sorenson C Kanavos P Karamalis M HTA in central and Eastern Europe: current status, challenges and opportunities Journal of Medical Device Regulation 2009 6 34 45

[B6] Kaló Z Landa K Doležal T Vokó Z Transferability of National Institute for Health and Clinical Excellence recommendations for pharmaceutical therapies in oncology to Central-Eastern European countries Eur J Cancer Care 2012 10.1111/j.1365-2354.2012.01351.x 22510226

[B7] Drummond M Griffin A Tarricone R Economic evaluation for devices and drugs–same or different? Value Health 2009 12 402 404 10.1111/j.1524-4733.2008.00476_1.x 19138306

[B8] Institut für Qualität und Wirtschaftlichkeit im Gesundheitswesen (IQWiG) 2011 General Methods Version 3.0 of 27.05. 2008, Germany https://www.iqwig.de/download/IQWiG_General_methods_V-3-0.pdf Accessed [11 June 2011]

[B9] National Institute for Health and Clinical Excellence (NICE), United Kingdom: Guide to the methods of technology appraisal ISBN: 1-84629-741-9. Issue date June 2008 2011 http://www.nice.org.uk/media/B52/A7/TAMethodsGuideUpdatedJune2008.pdf Accessed [11 June 2011]

[B10] National Institute for Health and Clinical Excellence (NICE), United Kingdom: Diagnostics Assessment Programme manual 2012 http://www.nice.org.uk/media/A0B/97/DAPManualFINAL.pdf Accessed [14 May 2012] 27466648

[B11] National Institute for Health and Clinical Excellence (NICE), United Kingdom: Medical Technologies Evaluation Programme. Methods guide 2012 http://www.nice.org.uk/media/3A6/09/MedicalTechnologiesEvaluationProgrammeMethodsGuideMarch2012.pdf Accessed [14 May 2012] 27905707

[B12] Adams ME McCall NT Gray DT Orza MJ Chalmers TC Economic analysis in randomised control trials Med Care 1992 30 231 238 10.1097/00005650-199203000-00005 1538611

[B13] Chiou CF Hay JW Wallace JF Bloom BS Neumann PJ Sullivan SD Yu HT Keeler EB Henning JM Ofman JJ Development and validation of a grading system for the quality of cost-effectiveness studies Med Care 2003 41 32 44 10.1097/00005650-200301000-00007 12544542

[B14] Evers S Goossens M de Vet H van Tulder M Ament A Criteria list for assessment of methodological quality of economic evaluations: Consensus on health economic criteria Int J Technol Assess Health Care 2005 21 240 245 15921065

[B15] Drummond MF Jefferson TO Guidelines for authors and peer reviewers of economic submissions to the BMJ. The BMJ Economic Evaluation Working Party BMJ 1996 313 275 283 10.1136/bmj.313.7052.275 8704542 PMC2351717

[B16] Szende A Mogyorósy Z Muszbek N Nagy J Pallos G Dózsa C Methodological guidelines for conducting economic evaluation of healthcare interventions in Hungary: a Hungarian proposal for methodology standards Eur J Health Econom 2002 3 196 206 10.1007/s10198-002-0109-6

[B17] Anis AH Gagnon Y Using economic evaluations to make formulary coverage decisions. So much for guidelines Pharmacoeconomics 2000 18 55 62 10.2165/00019053-200018010-00006 11010604

[B18] Augustovski F Iglesias C Manca A Drummond M Rubinstein A Martí SG Barriers to generalizability of health economic evaluations in Latin America and the Caribbean Region Pharmacoeconomics 2009 27 919 929 10.2165/11313670-000000000-00000 19888792

[B19] Ramsberg J Odeberg S Engström A Lundin D Examining the quality of health economic analyses submitted to the Pharmaceutical Benefits Board in Sweden Eur J Health Econom 2004 49 351 356 10.1007/s10198-004-0246-1 15452738

[B20] Public Health Resource Unit, United Kingdom: CASP 10 questions to help you make sense of economic evaluations 2010 [http://www.sph.nhs.uk/sph-files/Economic%20Evaluations%2010%20Questions.pdf]. Accessed [11 October 2010]

[B21] Health Economists’ Study Group How the group works? Website of the Health Economists’ Study Group 2012 http://www.hesg.org.uk/information.php Accessed [14 May 2012]

[B22] Gerkens S Crott R Cleemput I Thissen JP Closon MC Horsmans Y Beguin C Comparison of three instruments assessing the quality of economic evaluations: a practical exercise on economic evaluations of the surgical treatment of obesity Int J Technol Assess Health Care 2008 24 318 325 18601800 10.1017/S0266462308080422

[B23] Gonzalez-Perez JG Developing a scoring system to quality assess economic evaluations Eur J Health Econom 2002 3 131 136 10.1007/s10198-002-0100-2 15609137

[B24] Drummond MF O’Brien BJ Stoddart LG Torrance GW Critical assessment of economic evaluation. Methods for economic evaluation of health care programs 1997 2 Oxford University Press, New York

[B25] Halpern MT Luce BR Brown RE Geneste B Health and economic outcomes modeling practices: a suggested framework Value Health 1998 1 131 147 10.1046/j.1524-4733.1998.120131.x 16674361

[B26] EUnetHTA JA WP5: Relative Effectiveness Assessment (REA) of Pharmaceuticals 2012 http://www.eunethta.eu/upload/WP5/Link1.pdf Accessed [14 May 2012] 10.1017/S0266462314000592 25747560

[B27] Soto J Health economic evaluations using decision analytic modeling. Principles and practices—Utilization of a checklist to their development and appraisal Int J Technol Assess Health Care 2002 18 94 111 11987445

[B28] Boulenger S Nixon J Drummond M Ulmann P Rice S de Pouvourville G Can economic evaluations be made more transferable? Eur J Health Econom 2005 6 334 346 10.1007/s10198-005-0322-1 16249933

