# Supplementary material for: Attempt to increase the transparency of fourth hurdle implementation in Central-Eastern European middle income countries: publication of the critical appraisal methodology
Source: BMC Health Serv Res. 2012 Sep 21;12:332. doi: 10.1186/1472-6963-12-332 (PMC3465229; doi:10.1186/1472-6963-12-332)
Supplement: Additional file 1 — Hungarian critical appraisal checklist. [file 1472-6963-12-332-S1.doc]

| Completion guide: “Yes” answers indicate the correct methodology in the questionnaire. Not every question will be relevant for all economic evaluations. If a “yes” answer cannot be justified for a relevant question, the answer should be “no”. Explanations should be provided in the fourth column. Sub-questions in brackets also prompt explanations. | | | | |
| --- | --- | --- | --- | --- |
| Question | Answer | | | Explanation |
| 1. Economic Evaluation Checklist Questions | | | | |
|  | | | | |
| 1.1. Filter Questions | | | | |
| Did the evaluation present a well defined question? | yes [ ] | no [ ] | (If yes, please rephrase the question.) | |
| Did the evaluation give a comprehensive description of the competing alternatives? | yes [ ] | no [ ] | (If yes, please name the compared technologies.) | |
| 1.2. Research Question (relevance, comparator, financing protocol) | | | | |
| Is the selection of the comparator justified, considering the target indication, patient group, disease severity, therapeutic stage and the requested reimbursement category? | yes [ ] | no [ ] | (If yes, please clarify all details.) | |
| Does the economic evaluation adequately cover the full spectrum of indication submitted in the reimbursement application? | yes [ ] | no [ ] |  | |
| 1.3. Health Benefit | | | | |
|  | | | | |
| 1.3.1. Source of Scientific Evidence | | | | |
| Does the evaluation provide thorough evidence that the analyzed health technology is effective? | yes [ ] | no [ ] |  | |
| Does the evaluation provide thorough evidence that the comparator health technology is effective? | yes [ ] | no [ ] |  | |
| Was the search strategy for evidence supporting the effectiveness adequate and justifiable? | yes [ ] | no [ ] | (If yes, please indicate the methodology of literature review.) | |
| Were the search strategy and origin of quality of life or utility data presented? | yes [ ] | no [ ] | not relevant  [ ] |  |
| Were the references for health benefits indicated and attached (including data on file)? | yes [ ] | no [ ] |  | |
| If there is direct comparative trial, is the economic evaluation based on its results? (i.e. were results from direct comparison ignored in favour indirect comparison because the indirect comparions is more favorable?) | yes [ ] | no [ ] | not relevant  [ ] |  |
| Can we exclude that only favourable studies for the new health technology were taken into consideration? | yes [ ] | no [ ] |  | |
| 1.3.2. Evaluation of Relative Effectiveness in Case of Indirect Comparison | | | | |
| If indirect comparison was used, were the following assumptions of relative effectiveness calculation explained in detail? | yes [ ] | no [ ] | not relevant  [ ] |  |
| In the studies used as basis of indirect comparison calculations… | | | | |
| Was the methodology of the literature review to select the studies for the indirect comparison justified? (i.e. did they select even the less favourable studies?) | yes [ ] | no [ ] | not relevant  [ ] |  |
| Was the indirect comparison based on comparative studies and did those studies include the same comparator? | yes [ ] | no [ ] | not relevant  [ ] |  |
| Was the design of the selected studies similar, including the primary and secondary endpoints? | yes [ ] | no [ ] | not relevant  [ ] | (If not, please justify why the selected studies are acceptable and indicate the methodology of adjustment.) |
| Was the time horizon of the studies comparable? (i.e. did the assessment of the effectiveness – e.g. risk reduction – take place at the same time horizon?) | yes [ ] | no [ ] | not relevant  [ ] | (If not, please justify why the selected studies are acceptable and indicate the methodology of adjustment.) |
| Were the patient populations in the selected studies similar? (e.g. demographics, disease stage, and geographic location) | yes [ ] | no [ ] | not relevant  [ ] | (If not, please justify why the selected studies are acceptable and indicate the methodology of adjustment.) |
| Was the effectiveness measured in the same appropriate units in the selected studies? | yes [ ] | no [ ] | not relevant  [ ] |  |
| Were the absolute risk reduction (ARR) and relative risk reduction (RRR) also presented in each of the selected studies? | yes [ ] | no [ ] | not relevant  [ ] |  |
| Were the comparator groups at similar risk in the selected studies? (i.e., in the case of equivalent RRRs was ARR also the same?) | yes [ ] | no [ ] | not relevant  [ ] | (If not, please justify why the selected studies are acceptable and indicate the methodology of adjustment.) |
| Were all aspects of health benefit taken into consideration in the indirect comparison, including safety? (i.e. not only favourable aspects) | yes [ ] | no [ ] | not relevant  [ ] |  |
| 1.3.3. Magnitude of Health Benefit | | | | |
| Was every relevant and significant outcome and adverse event considered in the study when assessing health benefits? | yes [ ] | no [ ] | (If not, please indicate the justification) | |
| Do clinical trial data support the superior efficacy or improved side effect profile of the new health technology versus the comparator? | yes [ ] | no [ ] | not relevant  [ ] |  |
| Can we exclude the possibility that if there were no clinically significant efficacy or safety improvements based on clinical trials, they still projected long term incremental health benefits? | yes [ ] | no [ ] | not relevant  [ ] |  |
| Compared to short term outcomes (i.e. intermediate endpoints) is the difference in the long term outcomes similar and is the magnitude justifiable? | yes [ ] | no [ ] | not relevant  [ ] | (If not, please provide explanation.) |
| If survival data were previously presented in relevant clinical studies, were they taken into account when calculating health benefits? | yes [ ] | no [ ] | not relevant  [ ] |  |
| If quality of life or utility data were previously presented in clinical relevant studies, were they taken into account when calculating health benefits? | yes [ ] | no [ ] | not relevant  [ ] |  |
| If quality of life or utility data were derived from indirect literature references (i.e. not from clinical studies of the assessed technology), was the quality of data presented? | yes [ ] | no [ ] | not relevant  [ ] |  |
| Was the aggregation of different dimensions of effectiveness and adverse events adequate, and can the calculations be replicated? | yes [ ] | no [ ] | not relevant  [ ] | (How were the different dimensions of health benefits aggregated e.g. life years, QALY, other?) |
| 1.4. Cost | | | | |
| Was every relevant and significant outcome and adverse events considered in the cost calculation? | yes [ ] | no [ ] | (If not, please provide explanation.) | |
| Was the method of cost calculation clarified and the references for cost data presented? | yes [ ] | no [ ] |  | |
| Was the appropriate perspective employed in the economic evaluation? | yes [ ] | no [ ] | (If yes, please name the perspective.) | |
| Does the calculation of daily treatment cost correspond to the relevant dosage for the target indication in the local clinical practice? | yes [ ] | no [ ] | not relevant  [ ] |  |
| Is the calculation of the treatment duration justified in the target indication? | yes [ ] | no [ ] | not relevant  [ ] |  |
| Was the cost of pharmaceuticals calculated in full public price in the case of the investigated health technology, the comparator, and the adjunct therapies? | yes [ ] | no [ ] |  | |
| If the difference in the total cost of two therapies is smaller than the difference in the public price of the compared technologies, is the source of cost saving presented in the cost calculation? | yes [ ] | no [ ] | not relevant  [ ] |  |
| Overall is the cost calculation acceptable for the objectives of the economic evaluation? | yes [ ] | no [ ] |  | |
| 1.5. Time Horizon, Discounting | | | | |
| Was the time horizon adequately chosen in the analysis? | yes [ ] | no [ ] | not relevant  [ ] | (If yes, please indicate the time horizon.) |
| Are the time horizons of costs and health benefits equivalent in the analysis? | yes [ ] | no [ ] | not relevant  [ ] |  |
| Were resource use and health outcomes consequences adjusted for different times at which they occurred? (i.e. discounting with an appropriate discount rate) | yes [ ] | no [ ] | not relevant  [ ] |  |
| 1.6. Alternative Sections for Methodology | | | | |
|  | | | | |
| 1.6.1. Cost-Minimisation Analysis | | | | |
| Was the methodology of the cost minimisation analysis justified? | yes [ ] | no [ ] | not relevant  [ ] |  |
| Does reliable evidence support the equality of health benefit for the compared technologies? | yes [ ] | no [ ] | not relevant  [ ] |  |
| In case of statistically not significant non-inferiority, is the new technology expected to result in at least the same health benefit as the comparator? (i.e. if clinical trials had been many times larger, would the presumable conclusion still be non-inferiority?) | yes [ ] | no [ ] | not relevant  [ ] |  |
| 1.6.2. Cost Effectiveness Analyses | | | | |
|  | | | | |
| 1.6.2.1. Decision Tree Model | | | | |
| Was the model presented graphically? | yes [ ] | no [ ] | not relevant  [ ] |  |
| Were the relevant demographic and epidemiological data of the initial population presented? (e.g. age, gender, morbidity rates) | yes [ ] | no [ ] | not relevant  [ ] |  |
| Were the probabilities of each node presented including their references? | yes [ ] | no [ ] | not relevant  [ ] |  |
| Were the cost and health outcomes data at each endpoint presented transparently? | yes [ ] | no [ ] | not relevant  [ ] |  |
| 1.6.2.2. Markov Model | | | | |
| Was each Markov state clearly determined and presented? | yes [ ] | no [ ] | not relevant  [ ] |  |
| Was the structure of the model presented graphically, including transition routes between all health states? | yes [ ] | no [ ] | not relevant  [ ] |  |
| Was the length of Markov cycle appropriate for the disease progression and the technology? | yes [ ] | no [ ] | not relevant  [ ] |  |
| If reasonable, was half-cycle correction used? (if half-cycle correction is not necessary, the question is not relevant) | yes [ ] | no [ ] | not relevant  [ ] |  |
| Were the relevant demographic and epidemiological data of the initial population presented and justified including the distribution of the initial population among the health states? (i.e. is it possible to identify whether it is an incidence or prevalence model?) | yes [ ] | no [ ] | not relevant  [ ] |  |
| Were the transition probabilities for the model transparently presented including their reference? | yes [ ] | no [ ] | not relevant  [ ] |  |
| Were the cost and health outcomes data provided for each Markov state of the model? | yes [ ] | no [ ] | not relevant  [ ] |  |
| 1.6.2.3. Simulation Model | | | | |
| Is the application of simulation modelling justifiable? | yes [ ] | no [ ] | not relevant  [ ] | (If yes, what is the reason?) |
| Was the influence diagram graphically presented? | yes [ ] | no [ ] | not relevant  [ ] |  |
| Was the structure of the model transparent and traceable? | yes [ ] | no [ ] | not relevant  [ ] |  |
| Was every relevant and significant treatment option, outcome, and adverse event considered in developing the structure of the model? | yes [ ] | no [ ] | not relevant  [ ] |  |
| Were the relevant demographic and epidemiological data of the initial population and their references presented including the distribution of the initial population with regard to each baseline variable? (i.e., is it possible to identify the characteristics of the simulated patient group?) | yes [ ] | no [ ] | not relevant  [ ] |  |
| Were the applied baseline variables, individual patient routes and individual risks relevant for the local patient population? | yes [ ] | no [ ] | not relevant  [ ] |  |
| Were the values and the distribution of modeling variables provided including their references? | yes [ ] | no [ ] | not relevant  [ ] |  |
| Was the number of patients / simulation runs sufficient to produce consistent estimates? (if the model were run again, would we get very similar results?) | yes [ ] | no [ ] | not relevant  [ ] |  |
| 1.7. Decision Rule | | | | |
| Was the incremental cost effectiveness of the compared alternatives calculated? | yes [ ] | no [ ] | not relevant  [ ] | (If not, please provide explanation.) |
| Was the calculation of the ICER adequate and replicable? | yes [ ] | no [ ] | not relevant  [ ] |  |
| Is it possible to interpret the relative magnitude of the ICER from the payers’ decision-making perspective? | yes [ ] | no [ ] | not relevant  [ ] |  |
| 1.8. Sensitivity Analysis | | | | |
| Were sensitivity analyses undertaken? | yes [ ] | no [ ] | (If not, please provide explanation.) | |
| Was every uncertain parameter with strong influence on the results considered in the sensitivity analysis? | yes [ ] | no [ ] | not relevant  [ ] |  |
| If the time horizon of the analysis is crucial for the ICER, was sensitivity analysis undertaken for the time horizon? | yes [ ] | no [ ] | not relevant  [ ] |  |
| Is the conclusion of sensitivity analyses supported by adequate and transparent methodology? | yes [ ] | no [ ] | not relevant  [ ] |  |
| Were the conclusions of the sensitivity analysis summarised and presented? | yes [ ] | no [ ] | not relevant  [ ] | (If yes, please indicate the conclusions.) |
| Did the results of the sensitivity analysis support the robustness of the conclusion for the economic evaluation? | yes [ ] | no [ ] | not relevant  [ ] |  |
| 1.9. General Methodology: Adequacy and Transparency | | | | |
| Did the authors select the appropriate type and method of economic evaluation? (i.e. relevant for the disease progression and the assessed technologies) | yes [ ] | no [ ] | (If yes, which one?) | |
| If the economic evaluation is based on a model, has the model been attached to the dossier? | yes [ ] | no [ ] | not relevant  [ ] | (If not, please provide explanation.) |
| Does the economic evaluation cover each strength and dose of the new technology? (i.e. does it contain the less favourable strength and dose?) | yes [ ] | no [ ] | not relevant  [ ] |  |
| Is the presentation of the health economic evaluation proportionate? (i.e. is the results, conclusion, sensitivity analysis and interpretation section longer than the general introduction of the study?) | yes [ ] | no [ ] |  | |
| Are the limitations of the health economic evaluation presented? (i.e., are they not kept hidden?) | yes [ ] | no [ ] |  | |
| 1.10. Interpretation regarding the Economic Evaluation | | | | |
| Did the presentation and interpretation of the results of the economic evaluation cover every relevant aspect that needs to be considered when purchasing health care services? | yes [ ] | no [ ] |  | |
| Is the conclusion of the economic evaluation clear? | yes [ ] | no [ ] | (If yes, please rephrase the conclusion.) | |
| Were the conclusions of the economic evaluation justified by the evidence presented? | yes [ ] | no [ ] |  | |
| Can the results be applied here and now in the purchasing decisions of health care services for the population? | yes [ ] | no [ ] |  | |
| 2. Budget Impact Analysis Checklist Questions | | | | |
| With respect to the treatment, was the number of patients adequately calculated and was the method of calculation justified? | yes [ ] | no [ ] |  | |
| Does the calculation of daily treatment cost correspond to the expected dosage in the target indication and patient groups? | yes [ ] | no [ ] | not relevant  [ ] |  |
| Does the calculation of the length of treatment correspond to the expected therapeutic practice in the target indication and patient groups? | yes [ ] | no [ ] | not relevant  [ ] |  |
| Was sensitivity analysis undertaken for the budget impact as well? (e.g., number of patients, dose and length of treatment, market penetration, etc.) | yes [ ] | no [ ] |  | |
| Was the risk of off-label use considered in the budget impact analysis? | yes [ ] | no [ ] | not relevant  [ ] |  |
| Were the expected sales of the investigated technology appropriately estimated? | yes [ ] | no [ ] |  | |
| Was the net budget impact of the public financing appropriately estimated? | yes [ ] | no [ ] |  | |
| Was the location and time horizon of the budget impact and the potential savings adequately estimated? | yes [ ] | no [ ] | not relevant  [ ] |  |
| If the study declares savings, will they be realisable in real world for payers? | yes [ ] | no [ ] | not relevant  [ ] |  |
| If the study declares savings, will they be realisable in real world for providers? | yes [ ] | no [ ] | not relevant  [ ] |  |
| Will the reimbursement result in additional direct costs or financial burden for the patient? | yes [ ] | no [ ] | not relevant  [ ] |  |
